# What is Terminological Discipline and What is Not? Reply to Nadin (2012)

**DOI:** 10.1007/s10508-012-9957-z

**Published:** 2012-04-13

**Authors:** Sanne Nauts, Martin Metzmacher, Thijs Verwijmeren, Vera Rommeswinkel, Johan C. Karremans

**Affiliations:** Behavioural Science Institute, Radboud University Nijmegen, Montessorilaan 3, 6500 HE Nijmegen, The Netherlands

Nadin (2012) argue that our article, entitled “The Mere Anticipation of an Interaction with a Woman Can Impair Men’s Cognitive Performance” (Nauts, Metzmacher, Verwijmeren, Rommeswinkel, & Karremans, 2011) is, in fact, not about anticipation. Although we appreciate Nadin’s general concern about terminological discipline, we do not concur with his view that failing to take over Nadin’s (1987, 2003) definition of “anticipation” implies a lack of terminological discipline on our part.

Before discussing why we think that the processes we described in our research fit with common definitions of anticipation, we would first like to clarify some issues that we believe have not been captured correctly in Nadin’s commentary. First of all, it is suggested that our data were “interpreted as: the man’s mere thought of having sex with a woman affects his cognitive performance” and that men were “fantasizing…about sexual encounters.” Second, Nadin suggests that our research was about “the cognitive and emotional costs of impression management,” thereby equating a drop in cognitive performance with the “emotional costs” of an interaction. To avoid confusion, we would like to point out that, in our article, we do not suggest that men were thinking about sexual intercourse, and that we do not make any claims about the emotional costs of anticipating a mixed-sex interaction.

If we do *not* suggest that men were explicitly thinking about sex, or were feeling disconsolate, what processes *do* we expect to play a role in causing men’s cognitive performance to decline when anticipating an interaction with a woman? In our article, we suggests that impression management could be a key factor, in that men were preparing for the upcoming interaction (e.g., by thinking of what to say to the woman), or may try to “save” their resources so they could make a good impression in the upcoming interaction. These processes fit well with the definition of anticipation that we used (as “the act of looking forward” and “visualization of a future state”) (Merrian Webster Dictionary, 2002).

Whether or not our definition of anticipation exactly matches the definition as it is used in the field of anticipatory computing is, in our view, irrelevant.^1^ Different definitions of the same term can co-exist in different scientific subfields without causing any misunderstanding. Just to name a few examples: agency can refer to the instantiation of an object with an associated goal (in computer science: e.g., Luck & d’Inverno, 1995) or to dominant personality traits (in personality psychology; e.g., Wiggins, 1991). And, as a slightly more extreme example: rumination can refer to the digestion of grass in goats (in dairy science: e.g., Schirmann, von Keyserlingk, Weary, Veira, & Heuweiser, 2009) or to a focus on depressive thoughts in people (in psychology: e.g., Nolen-Hoeksema & Morrow, 1993). Thus, although we agree with Nadin (2012) that terminological discipline is of great importance, we do not agree with the notion that terminological discipline should entail homogenizing terminology across all scientific fields. In our view, a certain definition in a scientific subfield does not need to prescribe the exact usage of that term in every scientific endeavor.

## Footnotes

1 A literature search suggests that our research actually does follow a common definition of anticipatory behavior as used by others (i.e., others than Nadin) in the field of anticipatory computing, as “a process, or behavior, that does not only depend on past and present, but also on predictions, expectations, or beliefs about the future….Anticipation is really about the impact of a prediction or expectation on current behavior” (Butz, Sigaud, & Gerard, 2003). Our research, in which men were preparing for an upcoming interaction to be able to create a good impression on a woman later, fits this definition well.

## References

[CR1] Butz MV, Sigaud O, Gerard P, Butz MV, Sigaud O, Gerard P (2003). Anticipatory behavior: Exploiting knowledge about the future to improve current behavior. Anticipatory behavior in adaptive learning systems.

[CR3] Luck M, d’Inverno M, Lessor V (1995). A formal framework for agency and autonomy. Proceedings of the first international conference on multi-agent systems.

[CR2] Merriam-Webster Online Collegiate Dictionary (10th ed.). (2002). Retrieved from http://www.merriam-webster.com.

[CR4] Nadin M (1987). Mind: Anticipation and chaos.

[CR5] Nadin M (2003). Anticipation: The end is where we start from.

[CR6] Nadin, M. (2012). What is anticipation, and what is not? Comment on Nauts, Metzmacher, Verwijmeren, Rommeswinkel, and Karremans (2011) [Letter to the Editor]. *Archives of Sexual Behavior*, doi:10.1007/s10508-012-9926-6. 10.1007/s10508-012-9926-6 22434395

[CR7] Nauts, S., Metzmacher, M., Verwijmeren, T., Rommeswinkel, V., & Karremans, J. C. (2011). The mere anticipation of an interaction with a woman can impair men’s cognitive performance. *Archives of Sexual Behavior*, doi:10.1007/s10508-011-9860-z. 10.1007/s10508-011-9860-z PMC3394231 22042159

[CR8] Nolen-Hoeksema S, Morrow J (1993). Effects of rumination and distraction on naturally occurring depressed mood. Cognition and Emotion.

[CR9] Schirmann K, von Keyserlingk MAG, Weary DM, Veira DM, Heuweiser W (2009). Validation of a system for monitoring rumination in dairy cows. Journal of Dairy Science.

[CR10] Wiggins, J. S. (1991). Agency and communion as conceptual coordinates for the understanding and measurement of interpersonal behavior. In W. Grove & D. Cicchetti (Eds.), *Thinking clearly about psychology: Essays in honor of Paul E Meehl: Personality and psychopathology* (Vol. 2, pp. 89–113). Minneapolis, MN: University of Minnesota Press.

